# A quantitative *in vitro* collagen uptake assay

**DOI:** 10.1016/j.mex.2023.102288

**Published:** 2023-07-17

**Authors:** Sjors Maassen, Harry M. Warner, Pieter Grijpstra, Geert van den Bogaart

**Affiliations:** a Department of Molecular Immunology, Groningen Biomolecular Sciences and Biotechnology, University of Groningen, Groningen, the Netherlands; b Department of Medical Biology and Pathology, University Medical Centre Groningen, Groningen, the Netherlands

**Keywords:** Macrophages, Collagen, Extracellular matrix, A quantitative in vitro collagen uptake assay

## Abstract

Collagen remodelling is a vital process for embryonic development and homoeostatic maintenance of the adult body. Collagen remodelling is a complex process in fibroblasts, macrophages and other cells, whereby new collagen is secreted and polymerized into fibrils and old collagen is removed by proteolysis and endocytosis. Whereas the production of collagen is well-studied, the removal of collagen is less understood. In this protocol, we describe a method for the quantification of collagen uptake by cells. This protocol is based on the polymerisation of collagen type I-FITC conjugate in cell culture plate wells. Next, unpolymerized collagen is washed away and the cells are added in cell culture media. At this stage, they can be treated with inhibitors and/or stimulants if required. Afterwards, the cells are detached from the collagen using the protease accutase and the FITC signal is quantified using microscopy and/or flow cytometry.

• Easy-to-use protocol for the quantitative measurement of collagen uptake in cells.
• Cell detachment from collagen is quick and easy with accutase, even with strong adhering cells like macrophages.
• Downstream applications can be a wide selection of analysis techniques like microscopy, RNA- and protein isolation, and flow cytometry.

## Graphical abstract

**Figure:**
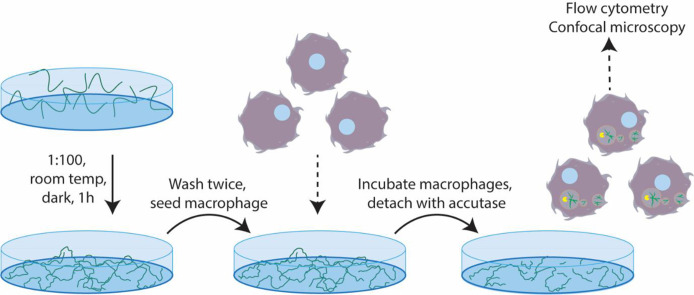

Specifications Table

**Table:** 

Subject area:	Immunology and Microbiology
More specific subject area:	*Collagen-immune interactions*
Name of your method:	*A quantitative in vitro collagen uptake assay*
Name and reference of original method:	https://www.sciencedirect.com/science/article/pii/S0891584922004622
Resource availability	N.A.

## Method

### Introduction

Collagen is the most abundant extracellular matrix component in the adult human body, contributing to roughly 25% of the body's total mass [1]. Furthermore, collagen fibrils are the largest protein polymers in vertebrates, with fibres up to 1 cm being previously observed [2]. Collagen has long been thought to be stable throughout the lifetime of adult humans and mice [3,4]. However recent work has pointed to the existence of a less-stable “sacrificial” collagen pool that is continually turned over in adult vertebrates [5]. Furthermore, extracellular collagen levels must be carefully controlled in the context of wound healing to prevent the emergence of fibrotic matrix. New collagen is mainly produced by fibroblasts, but also by other cells, and is well studied [6]. However, the removal of old collagen is less well studied, although recent work has highlighted the central role endosomes play in regulating collagen assembly to generate a functional ECM [7].

Macrophages are key drivers of collagen removal and remodelling. For example, macrophage-driven collagen remodelling plays a large role in wound healing [8], which is a complex multi-step process required for efficient closure of the wound and restoration of tissue homoeostasis. The uptake of excess collagen by the macrophages is an important step in this process. Insufficient clearance of collagen can result in scarring and fibrosis, in contrast, the overactivation of this process might lead to impaired wound healing. Fibroblasts can also ingest and degrade collagen [9], and this is thought to play similar roles in maintenance of tissue homoeostasis.

Collagen uptake and the cellular processes for its degradation are largely understudied. Following the uptake of collagen by cultured fibroblasts, collagen traffics to degenerative lysosomal compartments by the action of urokinase plasminogen activator receptor-associated protein (uPARAP/endo180) [10]. Tumour associated macrophages also degrade ingested collagen in lysosomal compartments, and this depends on the protease cathepsin B [11]. However, the mechanisms by which collagen reaches the lysosomes are still largely unclear, and the trafficking machinery which shuttles collagen to lysosomes (e.g. the soluble NSF attachment protein receptors, SNAREs) are only just beginning to emerge [7]. Similarly, while we know the receptors and integrins involved in the uptake of collagen (mannose receptor [12], CD11c [13], uPARAP/endo180 [10]) and ECM itself is able to signal to cells via integrins [14], however, we do not yet fully understand the signalling that regulates collagen uptake and processing. To address these questions, a method allowing for the quantification of collagen uptake by *ex vivo* cultured cells will be beneficial.

Here, we present a protocol allowing for the quantification of *in vitro* uptake of fibrous collagen type 1 by cultured cells at a single cell level by flow cytometry and microscopy. This assay is based on collagen type I from bovine labelled with a fluorophore (FITC). Despite originating from animal sources, FITC-labelled collagen has high biocompatibility with human cells [15], as collagen type I is highly conserved throughout the animal kingdom (Table 1). We recently developed this assay to investigate the uptake of collagen by human peripheral blood monocyte-derived macrophages [16]. However, this assay is widely applicable and can in principle also be used to study collagen uptake by other cell types and from different species, as well as to study the uptake of other ECM components. Thereby, this assay could contribute to our understanding of tissue homoeostasis and the disruption of this process in pathological tissues like fibrotic diseases and cancer. This assay could also be used for screening of compounds that affect endocytic collagen traffic for both laboratory and clinical settings.

**Table 1:** Conservation of human collagen type I in different species.

Species	Conservation of COL1A1	Conservation of COL1A2
*Bovine*	97.5%	92.9%
*Rattus norvegicus*	92.1%	91%
*Mus musculus*	91.7%	98.7%
*Danio rerio*	72.9%	–

## Method details

Step 1: Collagen polymerisation on tissue-culture plates

Materials and reagents

• Gibco DPBS, no calcium, no magnesium (Thermo Scientific, Cat no. 12559069)
• Gibco RPMI medium 1640(1X) (Fisher Scientific, Cat no. 11564456)
• Foetal Bovine Sera, Characterised (Hyclone Ge Healthcare, Cat no. 11521831)
• Gibco l-Glutamine(100X) (Fisher Scientific, Cat no. 15430614)
• Gibco anti-anti(100x) ((Fisher Scientific, Cat no. 15240–062)
• Collagen Type *I*−FITC Conjugate from bovine skin (Sigma Aldrich, Cat no. C4361–10ML)
• Corning Costar 24-well Clear TC-treated Multiple Well Plates (Fisher Scientific, Cat no. 10380932)
• CELLview™, petridish, TC, PS, 35 × 10 mm (Greiner Bio One, Cat no. 627870)
• Cover slips, 1–1/2 micro coverglass (Electron Microscopy Sciences, Cat no. 72230–01)
• Aluminium foil
• Paraformaldehyde (PFA), EM Grade (Aurion Cat no. 15710)
  ○ Comes in 16 percent, working solution is 4% in sterile 1X PBS

Step 1 Procedure

*Notes:*

(A) The media used in this protocol is optimal for human monocyte-derived macrophages, however, other cell types might require different media.
(B) FITC-collagen is a light sensitive molecule; try to keep in the dark as much as possible. Before starting the experiment, thaw the vial of 10 mL stock (∼1 mg/mL) on ice and aliquot stock into sterile tubes with volumes for one or two experiments (we use 100–200 µl into sterile PCR tubes) and store this at −20 °C – repeated freeze-thaw cycles should be avoided.
(C) When working with macrophages, one should note that these cells have repressed immune function on collagen [17].
(D) Collagen uptake can be dependent on phagocytosis. As a proof of concept, we showed that macrophages require dynamin for the uptake of collagen using the dynamin inhibitor dynasore. Macrophages were incubated with dynasore (full name: 3-hydroxynaphthalene-2-carboxylic acid (3,4-dihydroxybenzylidene)hydrazide; Abcam, Cat no. AB120192) at 0, 10 and 100 µM (from a 50 mM stock solution in anhydrous DMSO) while being cultured on collagen (24 h). Dynasore is a non-competitive reversible inhibitor of dynamin 1 and dynamin 2, the GTPases that are responsible for membrane scission in endocytosis. Whereas this completely blocks collagen uptake in human monocyte-derived macrophages, the dynasore control might not work for all cell types as not all forms of endocytosis require dynamin GTPases [18].

*Coating*:

1. Thaw an aliquot of FITC-labelled collagen on ice, keep on ice to prevent polymerisation.
2. In a sterile laminar flow hood, dilute the FITC-labelled collagen to 10 µg/mL in sterile 1X PBS (room temperature). Resuspend the dilution gently by pipetting (e.g. 10 mL of total volume is pipetted up and down twice with a 5 mL pipette).
  ○ For flow cytometry, add 250–300 µl FITC-labelled collagen to each well of a 24-well plate.
  ○ For live-cell imaging add 500–1000 µl FITC-labelled collagen to a CELLview, glass-bottom petri dish and gently swirl the liquid.
  ○ For immunofluorescence, sterilise glass coverslips by leaving them in 70% ethanol for 15 min, transfer a single coverslip to a 24-well plate well and wash them with sterile 1X PBS at room temperature for three times to wash away any residual ethanol. Then add 250–300 µl of FITC-labelled collagen.

In all cases, the entire bottom of the well should be covered. Cover the plates with aluminium foil and incubate them at room temperature for 1 hour to let the collagen polymerize.

3. Wash the wells twice with sterile 1X PBS to remove excess/unpolymerized collagen. Leave the wells in sterile 1X PBS at room temperature, in the dark, until use.
4. Culture the cells (see Seeding cells).

*Seeding cells*:

1. Remove PBS from the collagen-coated surfaces.
2. Add appropriate cell medium. For human monocyte-derived macrophages, use RPMI containing FBS (10%), glutamine (1X) and optionally anti-anti (1X): use 300–400 µl media for 24-well plates and 700–800 µl for CELLview.
3. Add 10^5^ cells to each 24-well. (i.e., 100 µl (10^5^) of cell suspensions (10^6^/mL)). For CELLview, add 3–5 × 10^5^ cells.
4. Incubate the cells in the incubator for 24 h. During this incubation, the cells will ingest the collagen.

*Optimisation item*:

• Our protocol uses a FITC-collagen concentration of 10 µg/mL, however, a higher volume and/or concentration will give a thicker and more connected collagen coating and/or more cross-linking of the collagen. This could affect the intake of collagen and hence influence the signal. Moreover, this makes the microscopy more challenging, because of two reasons: First, it increases the optical distance, and it might be not possible to get the cells in focus with high-magnification high-numerical aperture objectives. To overcome this problem, the sample can be ‘flipped’, thus inverted and put upside-down on another cover glass. Second, a thick layer of FITC-labelled collagen results in a high fluorescent background signal, potentially masking the fluorescence of the internalized collagen. This problem can be (at least partially) overcome by diluting the FITC labelled collagen with unlabelled collagen. For this dilution, prior to the second step of the protocol, 400 µl cold rat tail collagen I (A1048301; Thermo Fisher Scientific, Gibco) is added to a mix of 125 µl water, 15 µl 1 M NaOH and 60 µl 10 × PBS on ice. To this, a molar ratio of 1:100 FITC-labelled collagen is added. We recently published both these solutions [19].
• Depending on the migratory and endocytic capacity of the cells, varying seeding densities might affect collagen uptake.
• In macrophages, we have seen that LPS greatly increases the uptake of collagen [20], which in this cell type can serve as a positive control for collagen uptake.

Step 2: immunofluorescence and live-cell Imaging

Materials and reagents

• Immersion oil (Cargille, Cat no. 16237)
• RPMI 1640 without Phenol Red (Fisher Scientific, Cat no. 11564456)
• Nail polish
• Staining buffer (frozen aliquots)
  ○ Glycine 1.5 g (Bachem, Cat no. 4030676)
  ○ BSA 30 g (Fisher Scientific, Cat no. 11413164)
  ○ 1000 mL sterile 1X PBS (pH 7.5)
  ○ Filtered with a 0.22 µm Stericup (Millipore, Cat no. SCGPU02RE)
• Saponin in sterile 1X PBS (filtered) (Sigma Aldrich, Cat no. 47036–50G-F)
• Mounting media
  ○ 70% (w/v) Kaiser's glycerol gelatine for microscopy (Boom BV, Cat no. 55009242.0100)
  ○ 100 mM phosphate buffer (KH_2_PO_4_, Supelco, Cat no. 51004873.1000) at pH 8.0
  ○ 1 mM (*S*)−6-methoxy-2,5,7,8-tetramethylchromane-2-carboxylic acid (Trolox; Sigma-Aldrich, Cat no. 238813)
    ■ For 4 ml of 100 mM Trolox:
      • 100 mg Trolox
      • Add 860 µl of methanol and dissolve
      • Add 3077 µl of H2O (a precipitate will form)
      • Add 63 µl of 10 M NaOH (everything will dissolve, resulting in a light yellow solution)
  ○ Optional: 0.33 µg/ml DAPI (4′,6-diamidino-2-phenylindole dihydrochloride, Sigma-Aldrich Cat no. 32,670–5mg-F)
• Primary and fluorescently labelled secondary antibodies as appropriate

Step 2 procedure

*Live-cell imaging:*

*Notes:*

(A) For live-cell imaging, other media might be more suitable for other cell types, however media should be phenol red free.
(B) For live-cell imaging, make sure to pre-heat the microscope to 37 °C and, if possible, adjust CO_2_ to levels suitable for culture conditions.
(C) When using an oil lens, also warm up the oil to 37 °C. This will reduce thermal focus drift.
(D) To minimise phototoxicity, make sure to limit exposure time.
(E) For colocalization with other lysosomes, mitochondria or ER, it is also possible to add Lyso-, Mito- or ER-tracker.
1. Pre-warm phenol red free RPMI 1640 at 37 °C.
2. After 24 hour incubation in a CELLview, glass-bottom petri dish (see Materials and reagents Step 1), cells are inspected for viability under a light microscope.
3. In a sterile laminar flow hood; aspirate media from cells, wash once with 1 mL of pre-warmed phenol red free RPMI 1640 to remove the phenol red, and add 1 mL warm phenol red free RPMI 1640.
4. Optionally, to ensure proper coating before seeding, the wells can be imaged with a fluorescence microscope (Fig. 1A). We used a Zeiss LSM800 confocal with a DIC 63X oil lens (NA 1.4) with a CELLview, glass-bottom petri dish.

**Fig. 1:**
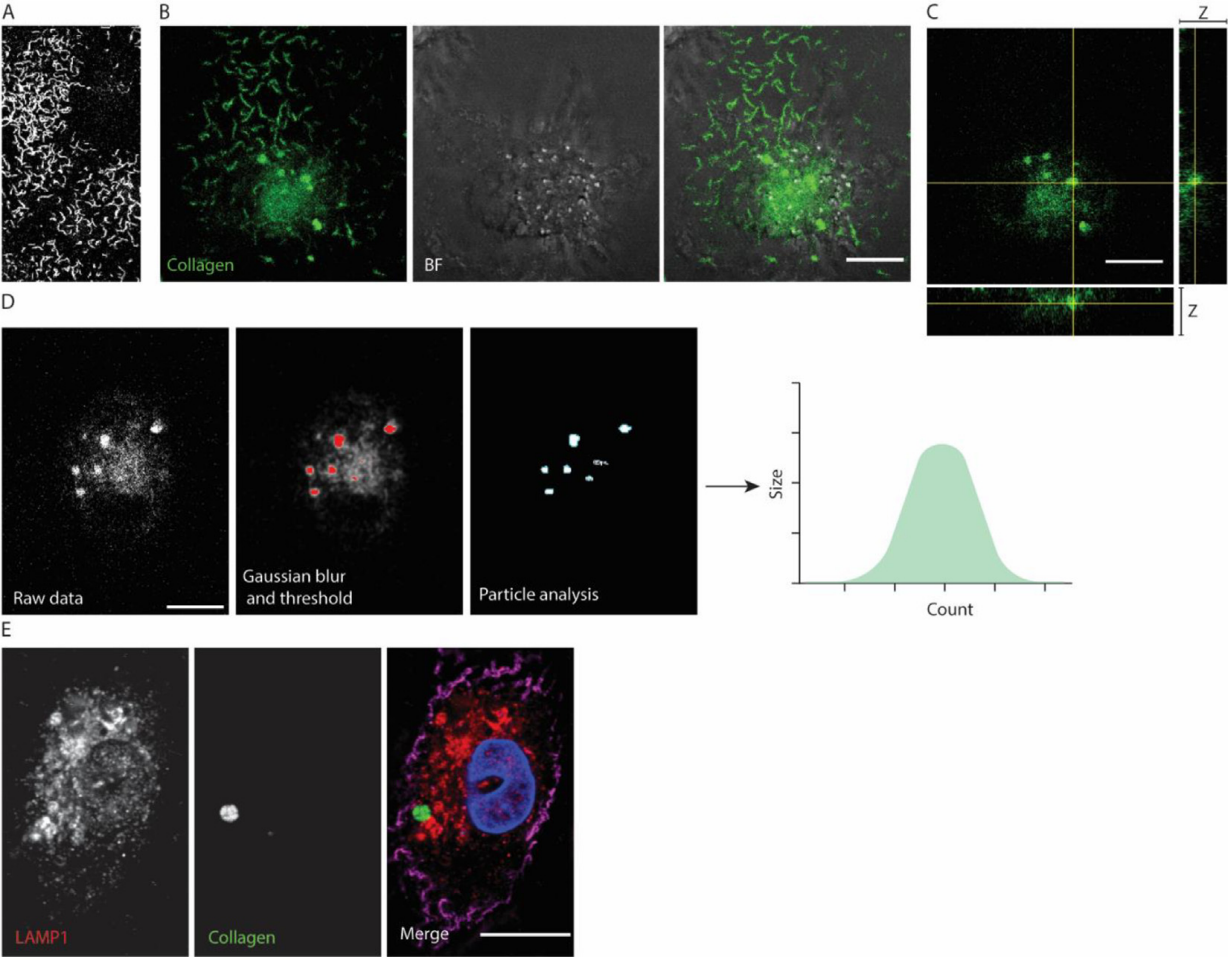
Microscopy of collagen coat and uptake by macrophages. (A) Fluorescence confocal microscopy image of FITC-collagen coat. (B) Confocal live-cell microscopy *z*-projection of human blood monocyte-derived macrophage on FITC-collagen coat showing intracellular accumulation of collagen. The *z*-step was 100 nm. (C) Single confocal micrograph (same cell as panel B). The optical plane was selected to visualize the intracellular collagen-containing compartment. (D) Automized quantification of the size and number of particles using a script in Fiji [21] (provided in supplementary information) that counts the particles above a fluorescence threshold. For our setup, the average number of collagen particles in macrophages is approximately 5–8 vacuoles per cell. (E) Confocal microscopy of human blood monocyte-derived macrophage with ingested FITC-collagen (green) and immunostained with lysosomal-marker LAMP1 (red), phalloidin (magenta) and DAPI (blue). *x*/*y* pixel steps were below 200 nm (i.e., diffraction limit of light microscopy). Scale bars are 10 µm.
5. To ensure the accumulation of collagen inside the cell, take confocal *z*-stacks of cells. The intracellular organelles containing ingested collagen look like distinct round structures (Fig. 1B-C).

*Immunofluorescence microscopy:*

*Notes:*

(A) Permeabilization of samples can also be done with Triton-X 100 (1–2%, v/v) prior to starting this protocol.
(B) We used saponin (0.1%, w/v) for our permeabilization, as it preserves the integrity of membrane vesicles like endosomes. Saponin needs to be present at all steps of the antibody labelling.
(C) Do not let glass coverslips with cells dry out, keep samples covered in PBS during the protocol.
(D) To reduce the FITC signal from non-internalized collagen, one can also culture cells in FITC collagen-coated 24-wells, detach them using accutase (see flow cytometry section below) and reseed them on glass coverslips with subsequent fixation.
1. For immunofluorescence: fix the cells with 4% (v/v) paraformaldehyde for 15 min at 4 °C, with 3 subsequent washes with sterile 1X PBS. Samples can be stored in PBS at 4 °C until staining.
2. Remove PBS from the fixed samples and add 300–400 µl staining buffer containing 0.1% saponin and incubate at room temperature for 30 min to permeabilize the cells.
3. Dilute primary antibodies in staining buffer with 0.1% saponin and incubate samples at 4 °C overnight for labelling with the primary antibodies. A typical dilution range that we apply for antibodies is between 100 and 200 fold volume/volume (final concentration about 5 µg/ml, see Optimisation item) .
4. Wash the samples three times with 1X PBS to remove unbound primary antibody.
5. Dilute secondary antibodies to 2.5 µg/ml (see Optimisation item, optionally add fluorescent phalloidin for visualisation of the F-actin, Fig. 1D) in staining buffer with 0.1% saponin and incubate samples at room temperature for 1 hour for labelling with secondary antibodies.
6. Wash the samples with 1X PBS three times to remove unbound secondary antibody.
7. Mount glass coverslips on microscope slides by adding 2.5 µl of mounting media (DAPI added optionally for visualisation of the nucleus, Fig. 1D) to the microscope slide. Before inverting coverslips on to the mounting media droplet, gently hold the side of the coverslip against a tissue to aspirate any surplus of liquid.
8. Leave the mounted samples in the dark for 1 h, so it cures slightly.
9. Seal the coverslips onto the microscope slide with nail polish by applying it over the edges of the coverslip onto the microscopy slide. This helps to preserve the sample longer for up to two weeks (store at 4 °C).
10. Image the samples with fluorescence microscopy.
11. For quantification, the number and size of the particles can be determined using a Fiji [21] script (provided as supplementary information) that automatically counts the particles above an automatically determined fluorescence threshold. Note that thresholding might differ for acquisition conditions and can influence the results of the analysis.

*Optimisation item*:

• Please note that antibody specificity might vary per concentration, cell type and fixation method.
• Collagen fibers can also be visualized using confocal reflection microscopy [22].
• In principle, the uptake of collagen can also be visualized using time-lapse live cell microscopy.

Step 3: Collagen uptake quantification by flow cytometry

Materials and reagents

• Stempro Accutase Dissociation Reagent (Fisher Scientific, Cat no. 11599686)
• 96-wells-plate, clear, polystyrene, V-bottom (Thermo Scientific, Cat no. 10462012)
• Fixable Viability eFLuor 780 (Thermo Fisher Scientific, Cat no. 15383562)

Step 3 procedure

*Detachment of cells:*

**Fig. 2:**
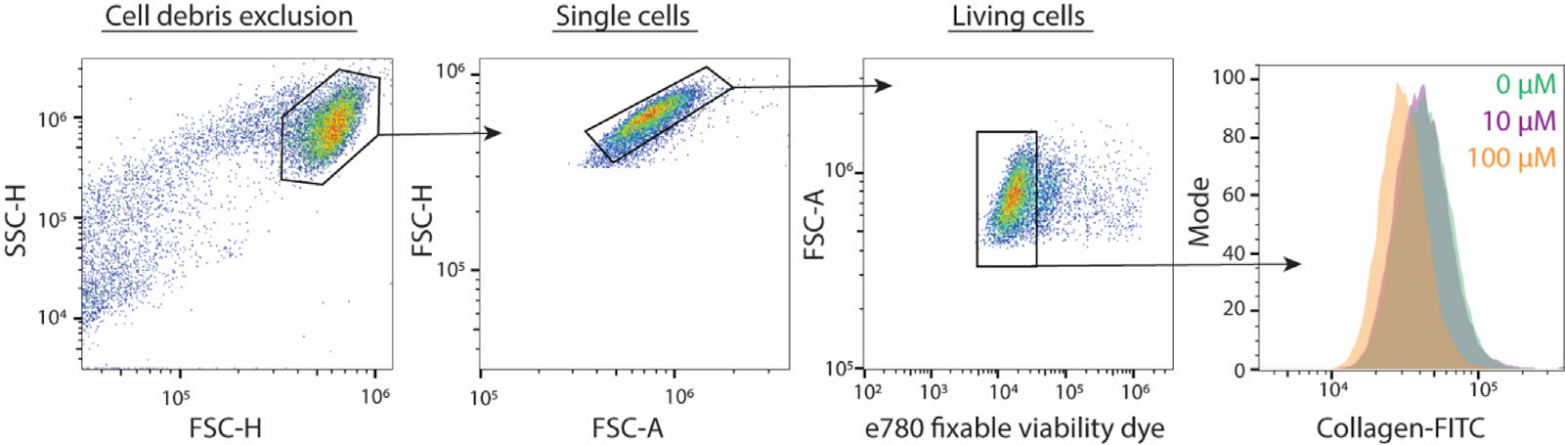
Example flow cytometry gating strategy of human monocyte-derived macrophages. Macrophages were cultured on FITC-collagen and stained with fixable e780 viability dye. Cells were selected on size (FCS: forward scatter; SSC: side scatter; H: height; A: area) to exclude cell aggregates. E780-negative cells were then selected for exclusion of dead cells. Cells were treated with 0, 10 and 100 µM dynasore as a control for uptake. The right-hand histogram shows the cells on the *y*-axis (normalized to mode) and the fluorescence intensity at the *x*-axis. Note that dynasore resulted in a lower fluorescence intensity (left-shifting of the histogram) in a dose-dependent manner.

*Notes:*

(A) The incubation time of the accutase supplier is used: 10 min in the incubator at 37 °C and 5% CO_2_. Detachment from the collagen can be followed by microscopy, as this results in a rounding of cell morphology.
(B) This protocol can also be carried out with trypsin instead of accutase, but we noticed a larger spread of data in flow cytometry. This could be related to the residual adhesion of collagen to cells. This problem might be resolved with prolonged incubations of trypsin. However, because trypsin cleaves many protein substrates, this might also reduce the effectivity of other stainings such as surface markers for further phenotyping of the cells.
(C) During step 7 of this protocol, there is an option to add flow cytometry antibodies and prolong the incubation time to 30 min. This flow cytometry antibody incubation should be preceded by a blocking step to prevent unspecific binding and block FC-receptors.
1. Remove media from the 24-well-plate and wash twice with 1X PBS
2. Add 200 µl accutase solution to the wells and gently swirl the plate to assure complete coverage of the well bottom.
3. Incubate the 24-well plate for 10 min in the incubator at 37 °C and 5% CO_2_ to let the cells detach.
  a. Check under the microscope, cells should round up upon detachment.
4. Pipette the accutase over the bottom of the well with a P1000, to release the remaining cells with shear force and transfer the content to a 96-well V-bottom.
5. To collect cells for staining and remove the free collagen, centrifuge (300 *xg*, 3 min, 4 °C) the 96-well plate with a plate centrifuge to collect the cells, decant the supernatant and briefly vortex the plate to resuspend the cell pellet in the V-bottom. The decanting does not remove all the liquid, but a small droplet (10–20 µl) remains. By vortexing, the cells are resuspended in this small volume.
6. Add 200 µl of 1X PBS to the 24-wells plate and repeat steps 4 and 5 to collect any cells that were left behind in the 24-well plate. Pool all cells in the 96-well plate. After this step, discard the 24-well plate
7. Dilute fixable viability eFluor 780 (1:1000 v/v) in cold PBS, vortex 10 s, and add 50–100 µl to the 96-well plate.
8. Incubate the plate 15 min at 4 °C in the fridge for the staining with eFluor 780.
9. Centrifuge (300 *xg*, 3 min, 4 °C) the 96-well plate with a plate centrifuge to collect the cells, decant the supernatant and briefly vortex the plate to resuspend the cell pellet in the remaining droplet in the V-bottom plate.
10. Add 50–100 µl 4% PFA to the wells for fixation.
11. Incubate the plate 15 min at 4 °C in the fridge to fix the cells.
12. To wash away residual PFA, centrifuge (300 *xg*, 3 min, 4 °C) the 96-well plate with a plate centrifuge, decant the supernatant and briefly vortex the plate to resuspend the cell pellet in the remaining droplet in the V-bottom plate.
13. Add 100–150 µl 1X PBS to dilute residual PFA.
14. Repeat step 12 and 13 for further washing.
15. Resuspend the cells in 100 µl 1X PBS and measure samples on plate-format flow cytometer (in our study CytoFlex S). Example gating strategy is depicted in Fig. 2.

*Optimisation item*:

• A potential problem is collagen detachment from the dish, particularly with shear force application. For this reason, we use enzymatic digestion of the collagen from the cells (accutase or trypsin) in our protocol

Additional Information

This technique can also be applied with other collagen types (e.g., type II and III collagen fibres, type IV network collagen, aggregated collagen), for example to distinguish preferences of collagen uptake among cell types.

## Declaration of Competing Interest

The authors declare that they have no known competing financial interests or personal relationships that could have appeared to influence the work reported in this paper.

## Data Availability

Data will be made available on request.
